# Identification of α-type subunits of the *Xenopus *20S proteasome and analysis of their changes during the meiotic cell cycle

**DOI:** 10.1186/1471-2091-5-18

**Published:** 2004-12-17

**Authors:** Yuka Wakata, Mika Tokumoto, Ryo Horiguchi, Katsutoshi Ishikawa, Yoshitaka Nagahama, Toshinobu Tokumoto

**Affiliations:** 1 Department of Biology and Geosciences, Faculty of Science, National University Corporation Shizuoka University, Shizuoka 422-8529, Japan; 2 CREST Research Project, Japan Science and Technology Corporation, Japan; 3 Laboratory of Reproductive Biology, National Institute for Basic Biology, Okazaki 444-8585, Japan

## Abstract

**Background:**

The 26S proteasome is the proteolytic machinery of the ubiquitin-dependent proteolytic system responsible for most of the regulated intracellular protein degradation in eukaryotic cells. Previously, we demonstrated meiotic cell cycle dependent phosphorylation of α4 subunit of the 26S proteasome. In this study, we analyzed the changes in the spotting pattern separated by 2-D gel electrophoresis of α subunits during *Xenopus *oocyte maturation.

**Results:**

We identified cDNA for three α-type subunits (α1, α5 and α6) of *Xenopus*, then prepared antibodies specific for five subunits (α1, α3, α5, α6, and α7). With these antibodies and previously described monoclonal antibodies for subunits α2 and α4, modifications to all α-type subunits of the 26S proteasome during *Xenopus *meiotic maturation were examined by 2D-PAGE. More than one spot for all subunits except α7 was identified. Immunoblot analysis of 26S proteasomes purified from immature and mature oocytes showed a difference in the blots of α2 and α4, with an additional spot detected in the 26S proteasome from immature oocytes (in G2-phase).

**Conclusions:**

Six of α-type subunits of the *Xenopus *26S proteasome are modified in *Xenopus *immature oocytes and two subunits (α2 and α4) are modified meiotic cell cycle-dependently.

## Background

Eukaryotic cells, from yeast to human, contain large nonlysosomal proteases called proteasomes [1]. The 26S proteasome is part of the ubiquitin-dependent proteolytic system, which regulates proteins through a mechanism of selective degradation [2-4]. The 26S proteasome is composed of a 20S proteasome as a catalytic core and regulatory particles at either end. The subunits of the 20S proteasome subunits can be classified into two families, α and β. In eukaryotes, the 20S proteasome contains seven α-type subunits and seven β-type subunits. The fourteen kinds of subunits are arranged in four rings of seven subunits and form an α7β7β7α7 structure [5].

Fully grown frog oocytes arrest in the late G2 phase of meiosis. Maturation-inducing hormone (MIH) acts on the oocytes, inducing final maturation and triggering germinal vesicle breakdown (GVBD), and the oocytes arrest again at the second meiotic metaphase until fertilization. The proteasomes are thought to be involved in regulating the maturation and fertilization of oocytes [6,7]. Previously we identified the proteasomal subunit modified during oocyte maturation in *Xenopus *and goldfish as α 4 [8,9]. In the present study, we cloned three unidentified α-type subunits of *Xenopus *and prepared antibodies for a total of five subunits. Using a set of specific antibodies, we analyzed changes in all α subunits composing the 26S proteasome during the meiotic cell cycle. We demonstrated that 6 of the subunits exist as a heterogeneous population in frog oocytes and identified another subunit in addition to α4 which was modified meiotic cell cycle dependently.

## Results and discussion

### Isolation and characterization of cDNA clones

**Figure 1:**
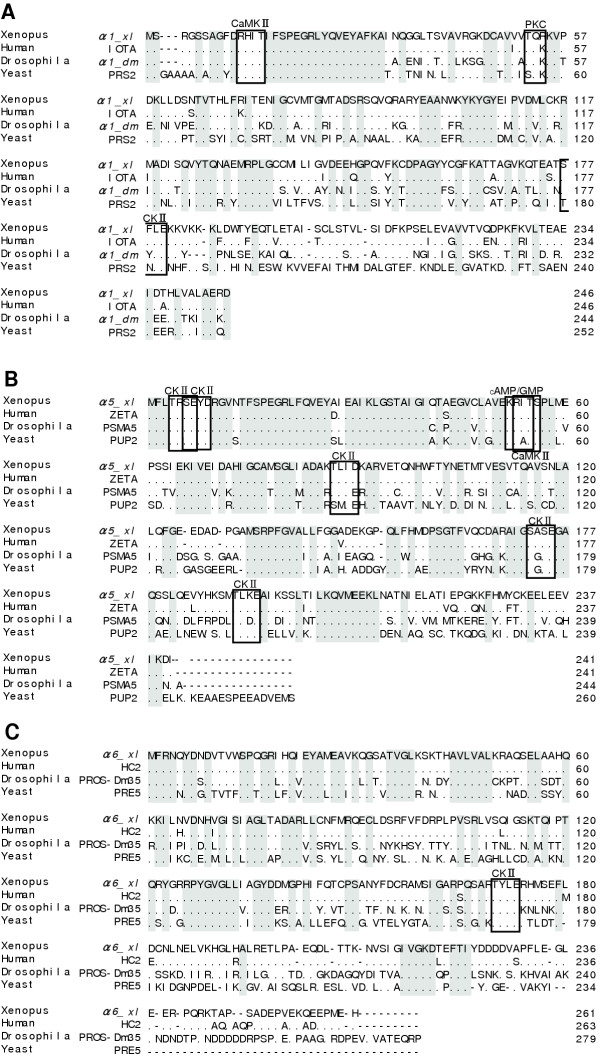
**Amino acid sequence comparison of the *Xenopus*, human, *Drosophila*, and Yeast α*1*, α*5 *and α*6 *proteasome subunits. **Amino acid sequence comparisons of α1 (A), α5 (B) and α6 (C) proteasome subunits are indicated. Matched sequences are boxed. Consensus sequences for calcium/calmodulin-dependent kinase II (CaMKII), cAMP/cGMP-dependent kinase (cAMP/cGMP), casein kinase II (CKII) and Ca^2+^-dependent kinase (PKC) are indicated. The numbers refer to the amino acid position at the end of each line.

**Figure 2:**
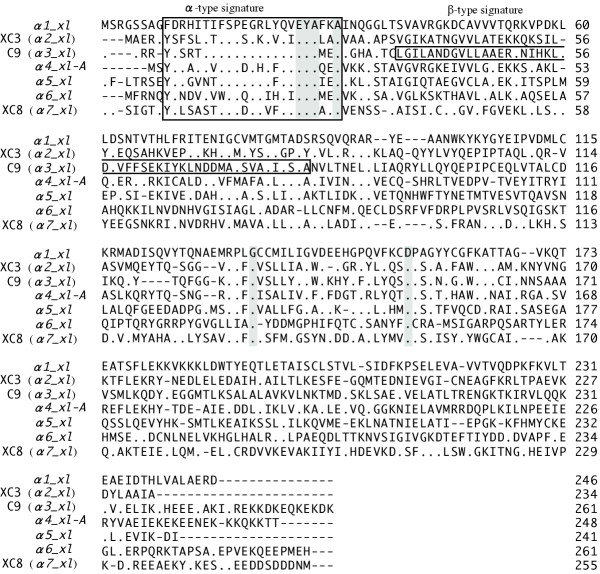
**Amino acid sequence comparison of the Xenopus proteasomal α subunits. **Matched sequences are boxed. The proteasomal α-type and β-type signatures were detemined by using the 'PROSITE' database [17] and are boxed. The numbers refer to the amino acid position at the end of each line.

A BLAST search of the *Xenopus *EST database was conducted using known proteasomal subunit α cDNAs. From the data for each subunit, full-length ORFs were obtained by PCR. The amplified cDNAs were 741, 726 and 786 bp long. The clones encode proteins of 246, 241 and 261 amino acid residues with a predicted molecular mass of 27463, 26402 and 29327 daltons, respectively (Fig. 1). Comparison of the amino acid sequence revealed that these molecules are highly homologous to the α1, α5 and α6 subunits in humans (overall identity 91.5–95.4%) [10,11], *Drosophila *(53.2–69.1%) [12,13] and yeast (53.2–61.7%) [14-16] (Fig. 2). Thus, we concluded that the cDNAs isolated in this study encode the α1, α5 and α6 subunits of the *Xenopu*s 20S proteasome. We named these clones *α1_xl, α5_xl *and *α6_xl *(α1, α5 and α6 subunits of *Xenopus laevis*) according to a systematic nomenclature [5]. Figure 2 represents a comparison of amino acid sequences predicted from cDNA sequences of α-type subunits of the *Xenopus *20S proteasome. Overall identity between the subunits was 25.1–38.4 %. A consensus sequence for α-type proteasomal subunits was conserved. Interestingly, a conserved sequence for β-type proteasomal subunits was found in the α3 subunit [17].

### Comparison of proteasomes purified from immature and mature oocytes

**Figure 3:**
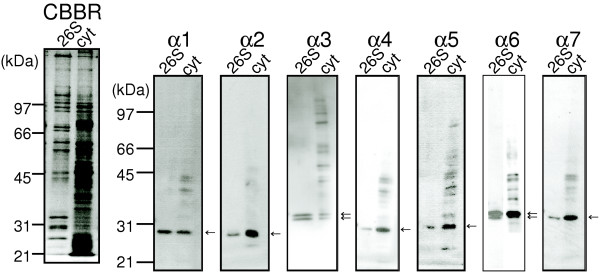
**Immunoblotting of the cytosol fraction and purified 26S proteasome. **The cytosol fraction and purified 26S proteasome were electrophoresed under denaturing conditions (10.0% gel) and stained with Coomassie Brilliant Blue (CBBR), or immunostained with antibodies for α subunits of the 20S proteasome. Lanes cyt and 26S indicate the cytosol fraction and the 26S proteasome from immature oocytes, respectively. Molecular masses of standard proteins are indicated at the left. Protein bands of each subunit are indicated by arrows.

**Figure 4:**
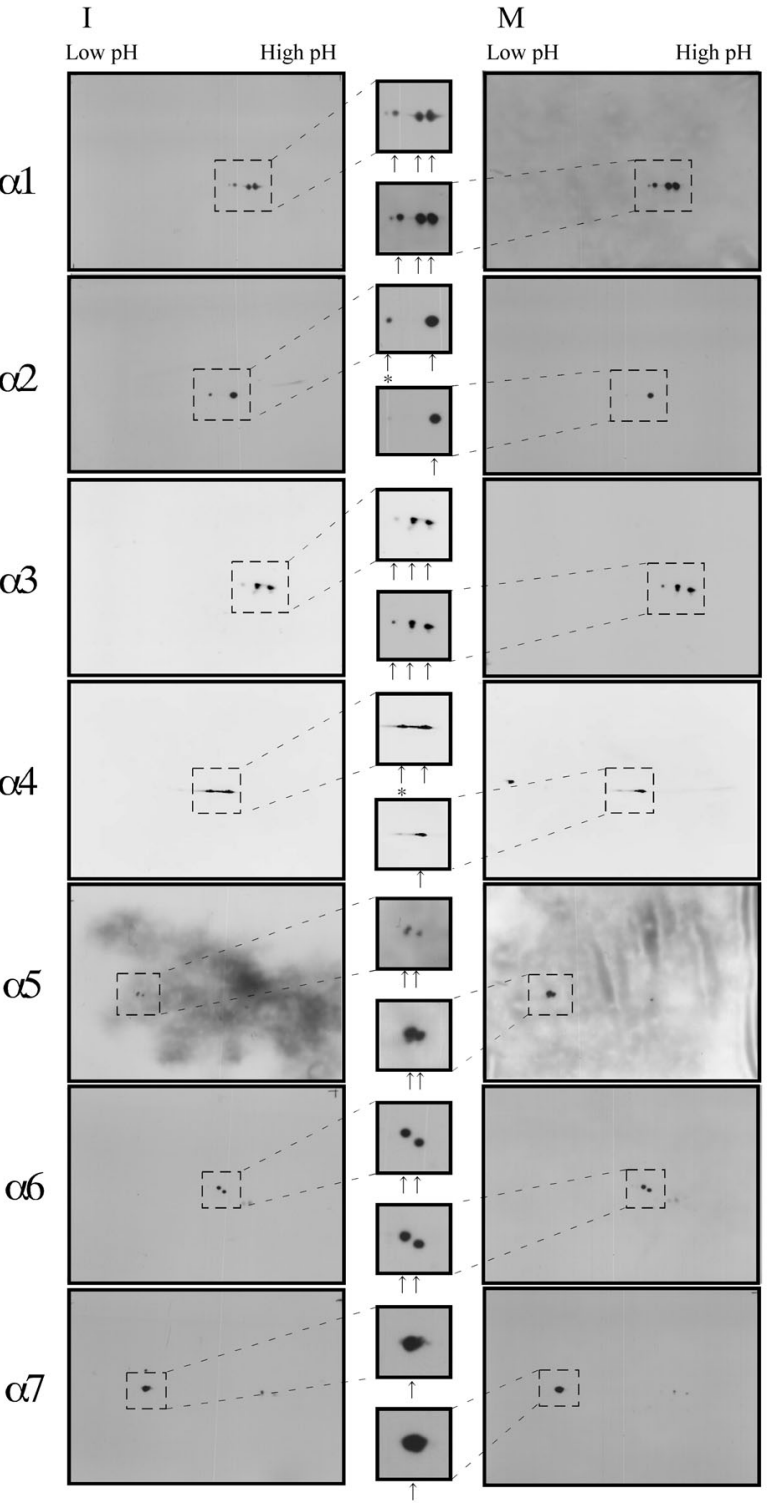
**2D-PAGE analysis of 26S proteasomes from immature and mature oocytes. **The 26S proteasomes from immature (I) and mature (M) oocytes were subjected to 2D-PAGE followed by immunostaining with polyclonal antibodies against each of the *Xenopus *20S proteasome subunits as indicated. The spots detected by each antibody are represented at high magnification and indicated by arrows. The spots differing between immature and mature oocytes are indicated by asterisks.

Polyclonal antibodies specific for five subunits (α1, α3, α5, α6, and α7) were raised against purified recombinant proteins. The specificity of the antibodies was examined by immunoblotting with the cytosol fraction and the purified 26S proteasome (Fig. 3). Each antibody preparation displayed a specific reaction for different polypeptides in both samples. Recombinant proteins from the cDNAs clearly cross-reacted with each antibody (data not shown). Thus, specific antibodies for each subunit were prepared. With these antibodies and previously described monoclonal antibodies for subunits α2 and α4 [18], changes to all α-type subunits during *Xenopus *meiotic maturation were analyzed. The modifications were demonstrated by 2D-PAGE (Fig. 4). The α7 subunit antibodies gave a single spot but all of the other antisera produced more than one spot, suggesting that the α1–α6 subunits undergo some type of modification in oocytes of *Xenopus *as demonstrated in other species [19,20]. A difference in the spots between the 26S proteasome from immature and mature oocytes was detected in the blots of subunits α2 and α4. In blots of α2 and α4, only a major spot was detected in the 26S proteasome from mature oocytes (in M-phase). It is suggested that the α4 subunit is phosphorylated in immature oocytes and dephosphorylated in mature oocytes [8]. Likewise, it is speculated that part of the α2 subunit is phosphorylated in interphase and dephosphorylated in metaphase. These results suggest that the subunits of 26S proteasomes are changed by meiotic cell cycle-dependent modifications. It can be speculated that these modifications are involved in the regulation of the meiotic cell cycle.

The modification of proteasomal subunits and factors interacting with proteasomes may be involved in the regulation of proteasome function [21]. By two-dimensional polyacrylamide gel electrophoresis, up to 20 different polypeptides were separated from the 20S proteasome which was shown to be composed of 14 gene products [22]. Furthermore, changes in proteasomal subunit composition under different physiological conditions and the likely existence of a different subpopulation of proteasomes have been reported [12,23]. All these results suggest that the subunit composition of proteasomes, and likely their activity, is under complex control *in vivo*. Some of these changes may be due to post-translational modifications of the proteasomal subunits. Regarding protein modification, there have been several reports about the phosphorylation of proteasomal subunits. Phosphorylated proteasomal subunits were detected in crude preparations from cultured *Drosophila *cells [22]. Several subunits of the 20S proteasome could be phosphorylated *in vitro *by a cyclic AMP-dependent protein kinase copurifying with the bovine pituitary 20S proteasome [24]. Castaño et al. [25] (1996) identified the CKII phosphorylating subunit and its phosphorylation sites as the C8 component (α7 subunit) and serine-243 and serine-250, respectively. CKII was also reported to phosphorylate the C2 component (α6 subunit) in rice [26]. The phosphorylation of subunits in the 26S proteasome *in vivo *was investigated using cultured human cells. Mason et al. [27] (1996) showed the phosphorylated subunits to be the C8 (α7 subunit) and C9 (α3 subunit) components in the 20S core, and the S4 (Rpt2p) subunit and several other components in regulatory particles [28]. Recent approaches have revealed post-translational modifications to many of the subunits. In the yeast 20S proteasome, the α2- and α4-subunits are phosphorylated at either a serine or threonine residue, and the α7-subunit is phosphorylated at tyrosine residue(s) [20]. In the human 20S proteasome, more than two spots were identified in all α-type subunits except α5 and phosphorylation of the α7-subunit at serine-250 was revealed [19]. However the sites and kinases responsible for the phosphorylation of the α2 and α4 subunits of the 20S proteasome have yet to be demonstrated. The modification of these proteins is one possible mechanism regulating the functions of the 26S proteasome during the meiotic cell cycle. Consensus sequences for phosphorylation sites are conserved in these subunits [8,29]. Cyclic-AMP dependent protein kinase is responsible for the G2/M and metaphase/anaphase transitions [30]. Calcium/calmodulin-dependent protein kinase II is shown to be involved in the exit from metaphase II arrest at fertilization in *Xenopus *[31]. It can be hypothesized that these kinases are involved in the regulation of 26S proteasome activity. The identification of kinases and the phosphorylation sites of the α2 and α4 subunits may reveal how the modification of proteasomal subunits is involved in controlling the cell cycle. Currently, we have identified one of the protein kinase for α4 subunit as Casein KinaseIα [32]. Possible regulation of 26S proteasome activity by this kinase is under investigation.

Recently, alternative subunits of proteasomes have been identified. In Drosophila where alternative α-type, β-type and 19S cap subunits are expressed from separate genes during spermatogenesis [33] and in Arabidopsis and rice where alternative isoforms of most proteasome subunits are differentially expressed from separate genes during development [34,35]. There are also examples of alternative β-type subunits in mammals (e.g., γ-interferon inducible "immunoproteasome" subunits β1i, β2i and β5i) [36]. Alternative subunits have yet to be identified in *Xenopus*, there is a possibility that the changes in the spots identified in this study may derive from differential expression of alternative subunits from paralogous genes.

## Conclusions

(1) cDNAs for three α-type proteasome subunits (*α1_xl, α5_xl *and *α6_xl*) of *X. laevis *were identified.

(2) Six subunits but not α7_XL are modified in immature oocytes in *X. laevis*.

(2) α2, α4_XLs are modified during the meiotic cell cycle in *X. laevis*.

## Methods

### Purification of proteasomes

Frogs (*Xenopus laevis*) were purchased from Jo-hoku Seibutsu Kyozai (Shizuoka, Japan) and maintained till used. 26S proteasomes were purified from immature oocytes and ovulated oocytes as described [37].

### Electrophoresis and immunoblotting

SDS-PAGE was carried out according to the method of Laemmli [38] (1970). 2D-PAGE (first dimension, NEPHGE; second dimension, SDS-PAGE) was carried out as described by O'Farrell et al. [39] (1977) using a precast polyacrylamide gel for NEPHGE (Immobiline Dry Strip pH3-10NL and pH6-11L for α4 subunit, Amersham biosciences) as reported [8]. Electroblotting and detection using antibodies were conducted as described [18].

### cDNA cloning and sequencing

Identification and sequence analysis of cDNAs. A BLAST search of the *Xenopus *EST database was conducted using known proteasomal subunit α cDNAs. From the data obtained for each subunit, independent sequences were linked and the full-length ORF sequences were eliminated(α1: BG347128 and CB558360, α5: BQ398972 and BJ043946, α6: BJ072624 and BJ091555). The specific primers for amplification of the full-length ORF were α1: 5'-GGAATTCCATATGTCTCGGGGATCTAGCGCG-3' and 5'-CCGCTCGAGGTCACGCTCAGCTAGTGCAAC-3', α5: 5'-GGAATTCCATATGTTCCTAACCCGCTCCGAG-3' and 5'-CCGCTCGAGGATGTCCTTAATAACTTCCTC-3', and α6: 5'-GGAATTCCATATGTTTCGCAATCAGTATG-3' and 5'-CCGCTCGAGGTGCTCCATAGGCTCCTCCTGC-3'), in which EcoRI (5'end) and XhoI (3'end) recognition sequence was added for cloning to the vector pET21a (Novagen). PCR was carried out using KOD DNA polymerase (TOYOBO) or LA taq DNA polymerase (TaKaRa), with *Xenopus *ovarian cDNA as a template, and the product was cloned to pET21a. The DNA sequencing was performed using a 377A DNA sequencer (Applied Biosystems). The sequences that include the full-length ORF identified here were deposited into GenBank (accession nos. AB164677, AB164678 and AB164679 for *α1_xl, α5_xl *and *α6_xl*, respectively). Pairwise comparisons of sequence homology were conducted using the Genetyx-Mac ver.12 computer program (Software Development, Tokyo, Japan).

### Production of recombinant proteins and preparation of antibodies

The recombinant proteins were produced in *E. coli *BL21 (LysE) and purified by SDS-PAGE as described [6]. Polyclonal antibodies specific for each subunit were raised against purified recombinant proteins according to a procedure described before using guinea pigs [40]. Anti serums, which recognize the bands of each subunit, were obtained.

## Abbreviations

bp, base pair; BLAST, basic local alignment search tool; cDNA, DNA complementary to RNA; EST, expressed sequence tags; kDa, kilodalton; NEPHGE, non-equilibrium pH gradient gel electrophoresis; PCR, polymerase chain reaction; SDS-PAGE SDS-polyacrylamide gel electrophoresis; 2D-PAGE, two-dimensional-PAGE.

## Authors' contributions

YW carried out cDNA cloning, expression of recombinant proteins, antibody production and 2D-PAGE analysis. MT and RH participated in cDNA cloning, expression of recombinant proteins. YN and KI participated in coordination of the study. TT carried out the protein purification and also participated in the design of the study and drafted the manuscript.
